# High Glucose-Induced Cardiomyocyte Death May Be Linked to Unbalanced Branched-Chain Amino Acids and Energy Metabolism

**DOI:** 10.3390/molecules23040807

**Published:** 2018-04-01

**Authors:** Xi Zhang, Qiuting Lin, Jiuxia Chen, Tingting Wei, Chen Li, Liangcai Zhao, Hongchang Gao, Hong Zheng

**Affiliations:** Institute of Metabonomics & Medical NMR, School of Pharmaceutical Sciences, Wenzhou Medical University, Wenzhou 325035, China; xizhang580@126.com (X.Z.); linqiuting2015@163.com (Q.L.); 18312910287@163.com (J.C.); weitingting992@163.com (T.W.); lichen2zh@163.com (C.L.); zhao_liangcai@126.com (L.Z.)

**Keywords:** BCAAs, cardiomyocyte death, diabetes, energy metabolism, high glucose, metabolomics

## Abstract

High glucose-induced cardiomyocyte death is a common symptom in advanced-stage diabetic patients, while its metabolic mechanism is still poorly understood. The aim of this study was to explore metabolic changes in high glucose-induced cardiomyocytes and the heart of streptozotocin-induced diabetic rats by ^1^H-NMR-based metabolomics. We found that high glucose can promote cardiomyocyte death both in vitro and in vivo studies. Metabolomic results show that several metabolites exhibited inconsistent variations in vitro and in vivo. However, we also identified a series of common metabolic changes, including increases in branched-chain amino acids (BCAAs: leucine, isoleucine and valine) as well as decreases in aspartate and creatine under high glucose condition. Moreover, a reduced energy metabolism could also be a common metabolic characteristic, as indicated by decreases in ATP in vitro as well as AMP, fumarate and succinate in vivo. Therefore, this study reveals that a decrease in energy metabolism and an increase in BCAAs metabolism could be implicated in high glucose-induced cardiomyocyte death.

## 1. Introduction

Diabetes mellitus (DM) is a common metabolic syndrome characterized by hyperglycemia due to insulin resistance or insulin secretion impairment [1]. In 2013, about 382 million people suffered from DM worldwide and this number is projected to increase to 592 million by 2035 [2]. In 2030, DM might become the seventh leading cause of death [3]. Of note, DM can cause multi-organ complications involving microvascular and macrovascular diseases [4]. The heart is not an exception in DM patients. Diabetic cardiomyopathy (DCM) has been the main cause of death in DM patients [5]. An epidemiological result reported that about 16.9% of DM patients suffered from DCM [6]. Cardiomyocyte death plays a key role in the development of DCM [7]. Up to now, a series of mechanisms underlying cardiomyocyte death has been proposed, such as autophagy [8], calpain activation [9], inflammation [10], endoplasmic reticulum stress [11], oxidative stress accumulation [12], glucotoxicity and lipotoxicity [13], intracellular angiotensin II production [14] and mitochondrial damage [15,16]. However, its pathogenesis is still far from being fully understood.

Metabolic dysfunction has also been associated with the onset and progression of DCM [17]. Energy metabolism is modified in the diabetic heart and characterized by enhanced fatty acid and reduced glucose consumption [18]. Yu et al. [19] found that a decreased expression of peroxisome proliferator-activated receptor delta (PPARδ), as a regulator of glucose and fatty acid metabolism, could be implicated in the development of DCM. Therefore, Ussher [20] suggested that the consequences of lipotoxicity or glucotoxicity play a key role in the prevention and treatment of DCM. In addition, perturbations in amino acid metabolism, especially branched-chain amino acids (BCAAs), have been associated with the development of DM [21] and heart disease [22]. Magnusson et al. [23] revealed that BCAAs could be an early link between DM and cardiovascular disease (CVD). Thus, we believe that exploring potential metabolic mechanisms is an important part for further understanding DCM pathogenesis. Metabolomics, as one of the omics techniques, attempts to detect a comprehensive set of metabolites in biological samples and analyze their changes related to pathophysiological states [24]. Metabolomics has been used as a promising tool for diagnosis, prognosis and pathophysiologic study of heart disease [25,26]. Nuclear magnetic resonance (NMR) spectroscopy is an attractive analytical platform for metabolomics research due to its advantages, such as simple sample preparation, fast analysis and high reproducibility. In our previous study, we have successfully applied an NMR-based metabolomic approach to elucidate metabolic mechanisms underlying diabetic complications related to kidney [27] and brain [28,29]. In the present study, we found that cardiomyocyte death was induced by high glucose both in vitro and in vivo studies. Therefore, we analyzed metabolic changes in high glucose-induced cardiomyocytes and the heart tissue of streptozotocin-treated diabetic rats, and attempted to explore its potential metabolic mechanisms using an NMR-based metabolomic approach. Our metabolomic results will provide metabolic information for further understanding the pathogenesis of DCM and will facilitate its prevention and treatment.

## 2. Materials and Methods

### 2.1. Animals

Sprague–Dawley (SD) rats at 8 weeks of age (male, body weight = 180–200 g) were purchased from the SLAC Laboratory Animal Co., Ltd. (Shanghai, China) and housed in a specific pathogen-free (SPF) colony with controlled temperature and humidity (temperature = 23 ± 2 °C; humidity = 55 ± 5%) as well as 12/12 h light/dark cycle (lights on at 8:00 a.m.) at the Laboratory Animal Center of Wenzhou Medical University (Wenzhou, China). All rats had free access to standard rat chow (Lab Diet 5001: 30% protein, 13% fat and 57% carbohydrate kcal/g) and tap water in this study. All animal procedures and their care were conducted in accordance with the Guide for the Care and Use of Laboratory Animals and approved by the Institutional Animal Care and Use Committee of Wenzhou Medical University (document number: wydw2016-0160). All experiments were reported according to the ARRIVE guidelines.

### 2.2. Streptozotocin-Induced Diabetic Rat Model

After 1-week of acclimation, all rats were weighted and randomly divided into control (Con, *n* = 10) and diabetic (DM, *n* = 10) groups. After a 12 h fasting, rats in the DM group were received intraperitoneal (i.p.) injection of streptozotocin (STZ, Sigma Aldrich, St. Louis, MO, USA) solution prepared in citrate buffer (0.1 M, pH 4.5) at a single dosage of 65 mg/kg of body weight. Rats in the Con group were injected with the same volume of sodium citrate. After 3 days of STZ injection, blood glucose level was measured using a handheld glucometer (One Touch Ultra, Lifescan, Milpitas, CA, USA) from a tail nick. The rat was defined as the DM rat when its blood glucose level >16.70 mmol/L.

### 2.3. Heart Tissue Collection and Preparation

In the previous study, Zhao et al. [30] reported that rats suffered from cardiac dysfunction after 8 weeks of STZ injection. In this study, therefore, rats at the end of 8 weeks after STZ injection (Con, *n* = 8; DM, *n* = 8, but one DM rat died from ileus) were randomly selected, overnight fasted and sacrificed by rapid decapitation to avoid stress responses. The whole heart tissues were collected after perfusion, snap-frozen in liquid nitrogen, and stored at −80 °C until use. The frozen heart tissues were weighed into an Eppendorf tube and ground using an electric homogenizer (FLUKO, Shanghai, China). Then, ice-cold methanol (4 mL/g) and distilled water (0.85 mL/g) were added and homogenized by vortex. Next, ice-cold chloroform (2 mL/g) and distilled water (2 mL/g) were added consecutively and mixed by vortex. After standing on ice for 15 min, the mixture was centrifuged at 1000× *g* at 4 °C for 15 min. The supernatant was transferred into a fresh Eppendorf tube, lyophilized for 24 h, and stored at −80 °C until use.

### 2.4. Histopathological Analysis

The change of myocardial structure and cytoplasmic distribution was evaluated by Haematoxylin-Eosin (HE) staining as described by Yu et al. [31]. Two normal rats (Con) and two diabetic rats (DM) were overnight fasted and sacrificed by rapid decapitation. All of the heart tissues were rapidly dissected, fixed overnight with 10% buffered neutral formalin, and embedded in paraffin after ethanol washing. The paraffin-embedded heart tissue was sliced into 5 μm sections, deparaffinised in xylene and ethanol series, and then washed in purified water. Finally, paraffin sections were stained according to standard protocols from the HE stain kit (Beyotime Institute of Biotechnology, Shanghai, China). The Eclipse 80i Fluorescence microscope (Nikon, Tokyo, Japan) was applied to capture the stained cardiac sections at 400× magnification. Then, we randomly selected 6 different sections to calculate percentages of normal myocardial cells in the total section area.

### 2.5. Cell Culture and Treatment

Embryonic rat heart-derived H9C2 cells were obtained from the Shanghai Institute of Biochemistry and Cell Biology (Shanghai, China) and cultured in DMEM medium (GIBCO, Thermo Fisher, Waltham, MA, USA) supplemented with 10% fetal bovine serum (FBS, Invitrogen, Carlsbad, CA, USA) and 1% penicillin-streptomycin (Invitrogen) in a humidified atmosphere of 5% CO_2_ at 37 °C. When the cells reached approximately 80% confluences, the cells were washed with Hank’s Buffered Salt Solution (HBSS) containing calcium and magnesium and exposed to either normal glucose (NG, 5.5 mM) or high glucose (HG, 33 mM) for 48 h [32].

### 2.6. Flow Cytometry Analysis

The cell apoptosis and necrosis were measured using an Annexin V-FITC Apoptosis Detection Kit according to the manufacturer’s instruction (BD, Biosciences, San Jose, CA, USA). In brief, the cells were seeded in 6-well plates at a density of 1 × 10^5^ cells/well, washed by cold PBS twice, and then incubated with 5 μL fluorescein isothiocyanate (FITC)-annexin V and 3 μL propidium iodide (PI)at the room temperature for 10 min in the dark [33]. Then, cellular fluorescence was detected by flow cytometry analysis (FACS Calibur^TM^, BD, Biosciences, San Jose, CA, USA) and analyzed with Flowjo software (version 9.3.2, BD, Biosciences, San Jose, CA, USA).

### 2.7. Hoechst Staining Analysis

Apoptotic cells were measured by Hoechst 33342 staining under a fluorescence microscope. Briefly, the cells were seeded in 6-well plates, stained with Hoechst 33342 for 5 min in the dark, and washed with PBS three times [34]. Then, the cells were observed using a fluorescence microscopy (IX73, Olympus, Tokyo, Japan). The experiment was performed in triplicate.

### 2.8. Intracellular Metabolite Extraction

The cells were harvested and washed three times with ice-cold PBS, and then methanol-chloroform-water extraction was conducted as described previously [35]. Briefly, the cells were resuspended in 500 μL of ice-cold methanol-chloroform (2:1, *v*/*v*) mixture, transferred into a 1.5 mL Eppendorf tube, and vortexed thoroughly. The tube was incubated on a mixer for 10 min at 4 °C. Then, 250 μL of ice-cold chloroform-water (1:1, *v*/*v*) was added and mixed using a vortex mixer. The mixture was ultrasonicated on ice for 10 min and centrifuged at 10,000× *g* for 20 min at 4 °C. The supernatant was extracted, lyophilized and stored at −80 °C until analysis.

### 2.9. Extracellular Metabolite Extraction

Metabolites in the conditioned culture medium were extracted using a modified Bligh–Dyer method [36]. In brief, 1 mL of culture medium was transferred from each culture flask into a 15 mL centrifuge tube. Then, 3 mL of ice-cold methanol-chloroform (2:1, *v*/*v*) mixture was added and followed by 1 mL of ice-cold chloroform. The mixture was centrifuged at 10,000× *g* for 20 min at 4 °C, and the upper phase fraction was lyophilized and stored at −80 °C for further analysis.

### 2.10. ^1^H-NMR-Based Metabolomic Analysis

The lyophilized extracts from heart tissue, cell and media samples were respectively dissolved in 500 μL D_2_O containing sodium trimethylsilyl propionate-d_4_ (TSP, 0.42 mM) and transferred to a 5-mm NMR tube for NMR analysis. ^1^H-NMR spectra were recorded on a Bruker AVANCE 600 MHz NMR spectrometer (Bruker BioSpin, Rheinstetten, Germany) with a 5-mm TXI probe at 298 K. A standard single-pulse sequence, ‘ZGPR’, with water signal pre-saturation was used to acquire ^1^H-NMR spectra. Moreover, typical acquisition parameters were set as follows: scans = 256; spectral width = 12,000 Hz; data points = 64 K; relaxation delay = 6 s; acquisition time = 2.65 s per scan.

^1^H-NMR spectra were manually phase- and baseline-corrected and referenced to the methyl peak of lactate (CH_3_, 1.33 ppm) [37] in Topspin software (version. 2.1, Bruker Biospin, Rheinstetten, Germany). The ‘icoshift’ procedure was used to align NMR spectra in MATLAB (R2012a, The Mathworks Inc., Natick, MA, USA) [38]. The spectral regions from 0.0 to 9.0 ppm excluding residual water signal (5.5–6.0 ppm) were subdivided and integrated to binning data with a size of 0.01 ppm for further multivariate analysis. The NMR signals were assigned using the Chenomx NMR suite 7.0 (Chenomx Inc., Edmonton, AB, Canada) and the Human Metabolome Database [39] as well as the reported data on heart tissue [40]. For metabolite quantification of heart tissue, its peak area was manually integrated using Topspin software and calculated according to its peak area by reference to the internal TSP concentration and the corresponding fresh weight tissue (FWT). The concentration of metabolite was expressed as μmol/g FWT. For cell and medium samples, each integrated peak was normalized to the total spectral area for minimizing differences in cell number and solvent in the culture medium. The level of metabolite was calculated according to its peak area by reference to the internal TSP concentration and expressed as relative units (r.u.).

### 2.11. Multivariate Analysis

Orthogonal partial least squares-discriminant analysis (OPLS-DA) was performed to examine the metabolic difference between the two groups using SIMCA 12.0 software (Umetrics, Umeå, Sweden). Prior to OPLS-DA, metabolomic data were Pareto-scaled, and a leave-one-out cross-validation (LOOCV) method was performed, where two parameters were calculated to evaluate the performance of OPLS-DA model: R^2^, the explained variance of the model; Q^2^, the predictive capability of the model. In general, these two parameters close to 1.0 represent a good model. Moreover, a CV-ANOVA method was used to assess model reliability [41]. The S-Plot was generated from the OPLS-DA model to identify the specific metabolites that mainly contributed to class discrimination.

### 2.12. Statistical Analysis

In this study, all rats were randomly assigned to the experimental procedures including housing and feeding and STZ injection. All samples were randomly analyzed by NMR spectroscopy. Analysis of variance (ANOVA) was conducted using Student’s *t*-test in SPSS software (version 13.0, SPSS Inc., Chicago, IL, USA), and a *p*-value < 0.05 was considered as a statistically significant difference.

## 3. Results

### 3.1. High Glucose Promotes Cardiomyocyte Death In Vitro and In Vivo

**Figure 1:**
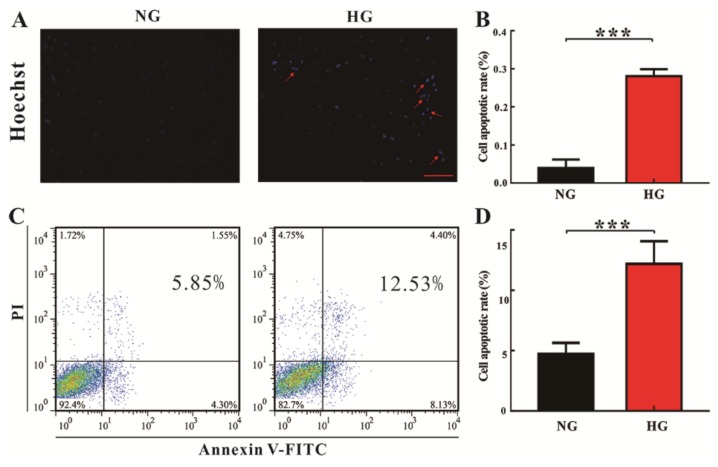
Changes in H9c2 cell number under normal glucose (NG, 5.5 mM) and high glucose (HG, 33 mM) conditions: (**A**) representative graphs of Hoechst 33342 staining of cell apoptosis after NG or HG exposure for 48 h; (**B**) the percentage of H9c2 cell apoptosis based on Hoechst 33342 staining (Means ± SE, *n* = 3); (**C**) representative graphs of flow cytometry analysis of cell apoptosis after NG or HG exposure for 48 h; in these four quadrants, viable cells are located in the left lower quadrant (FITC−/PI−), early apoptotic cells in the right lower quadrant (FITC+/PI−), late apoptotic cells in the right upper quadrant (FITC+/PI+), and necrotic cells in the left upper quadrant (FITC−/PI+); (**D**) the percentage of H9c2 cell apoptosis based on flow cytometry analysis (Means ± SE, *n* = 3); significant level: *** *p* < 0.001.

**Figure 2:**
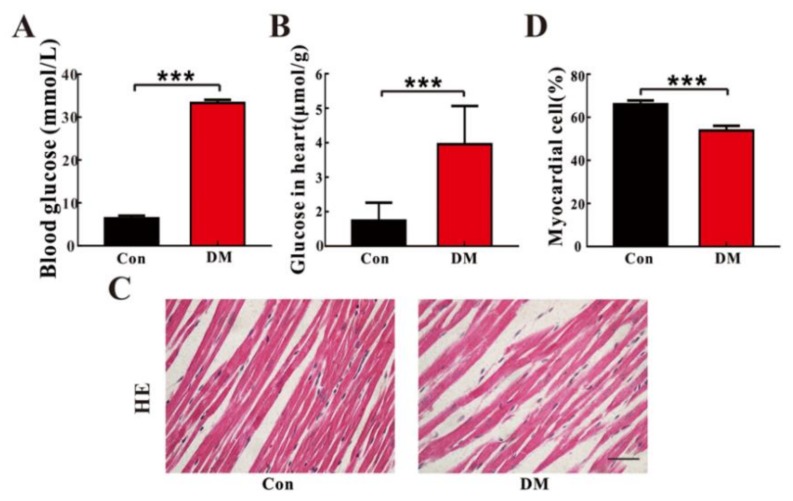
Changes in cardiomyocyte number in streptozotocin (STZ)-induced diabetic (DM) and normal control (Con) rats: the levels of glucose in the serum (**A**) and the heart (**B**) of DM and Con rats (Means ± SE, *n* = 6); (**C**) representative graphs of Haematoxylin-Eosin (HE) staining of heart tissues in Con and DM rats; (**D**) percentage of normal myocardial cell calculated from the HE staining (Means ± SE, *n* = 6); significant level: *** *p* < 0.001.

High glucose (HG)-induced H9c2 cell changes were examined by using Hoechst nuclear staining and flow cytometry analysis, and the corresponding results were illustrated in Figure 1. The Hoechst nuclear staining is commonly used to detect condensed chromatin in apoptotic cells. Thus, results demonstrate that apoptotic cells had a significantly higher condensation of nuclei under HG conditions than normal glucose (NG) conditions (Figure 1A,B; *p* < 0.0001). Consistent with the Hoechst staining results, we also found that the death rate of H9c2 cells was significantly increased under the HG conditions as compared with the NG level (Figure 1C,D; *p* < 0.0001). To verify HG-induced cardiomyocyte changes in vivo, heart tissues in STZ-treated T1D (type 1 diabetes, DM) rats at eight weeks of age were analysed by histopathological examination. As expected, the levels of glucose in the serum and the heart were significantly higher in DM rats at eight weeks of age than that in age-matched normal control (Con) rats, as shown in Figure 2A,B, respectively. In addition, compared with Con rats, the arrangement and structure of cardiomyocytes were obviously damaged in DM rats at eight weeks of age (Figure 2C). Figure 2D also shows that the percentage of normal cardiomyocytes was significantly reduced in DM rats relative to Con rats (*p* < 0.001). Taken together, our results based on in vitro and in vivo evidence reveal that HG can promote cardiomyocyte death.

### 3.2. High Glucose Induces Metabolic Changes in Cardiomyocytes

**Figure 3:**
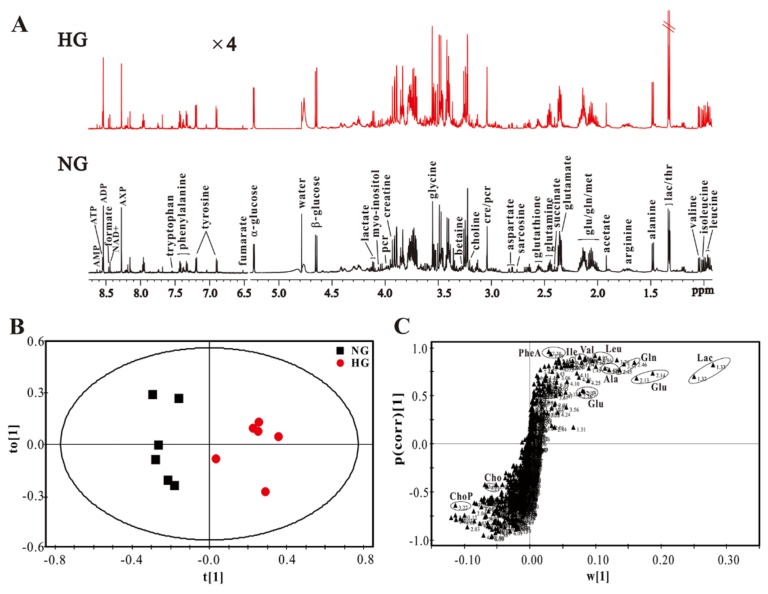
NMR-based H9c2 cell metabolomic analysis: (**A**) typical 600 MHz ^1^H-NMR spectra obtained from the intracellular extracts under high glucose (HG, 33 mM) and normal glucose (NG, 5.5 mM) conditions; OPLS-DA scores plot (**B**) and its corresponding S-plot (**C**) based on the metabolic profiles of H9c2 cells. Metabolite: ChoP, choline phosphate; Cho, choline; PheA, phenylalanine; Glu, glutamate; Ile, isoleucine; Val, valine; Leu, leucine; Gln, glutamine; Lac, lactate.

Figure 3A shows typical ^1^H-NMR spectra in the intracellular extracts of H9c2 cells cultured under NG and HG conditions. We identified a series of intracellular metabolites, involving amino acid metabolism (leucine; isoleucine; valine; phenyalanine; tryptophan; tyrosine; arginine; sarcosine; glycine), energy metabolism (lactate; alanine; acetate; glutamate; glutamine; succinate; aspartate; fumarate; creatine; creatine phosphate; NAD^+^, nicotinamide adenine dinucleotide; ADP, adenosine diphosphate; ATP, adenosine triphosphate; AMP, adenosine monophosphate), membrane metabolism (choline; choline phosphate), osmolyte (betaine; myo-Inositol) and other metabolites (glutathione; formate).

Then, OPLS-DA was performed to identify the differences in metabolic patterns between H9c2 cells cultured under NG and HG conditions. It can be seen from Figure 3B that metabolic patterns were obviously different between the two groups. Moreover, the corresponding S-plot of OPLS-DA shows that the metabolic difference was ascribed to increases in lactate, glutamine, alanine, phenylalanine, valine, leucine and isoleucine levels as well as decreases in choline and choline phosphate levels in H9c2 cells under HG conditions, relative to NG conditions (Figure 3C).

The extracellular extracts of H9c2 cells under NG and HG conditions were also analyzed by an NMR-based metabolomic method, and typical ^1^H-NMR spectra were shown in Figure S1A. A series of metabolites were assigned, such as leucine, isoleucine, valine, phenyalanine, tryptophan, tyrosine, lactate, alanine and acetate. The result based on OPLS-DA displays a clear separation between NG and HG culture media (Figure S1B). It can be seen from Figure S1C that this separation could be attributed to increases in lactate, choline phosphate and acetate as well as decreases in leucine, isoleucine and valine in HG culture medium as compared with NG culture medium.

**Figure 4:**
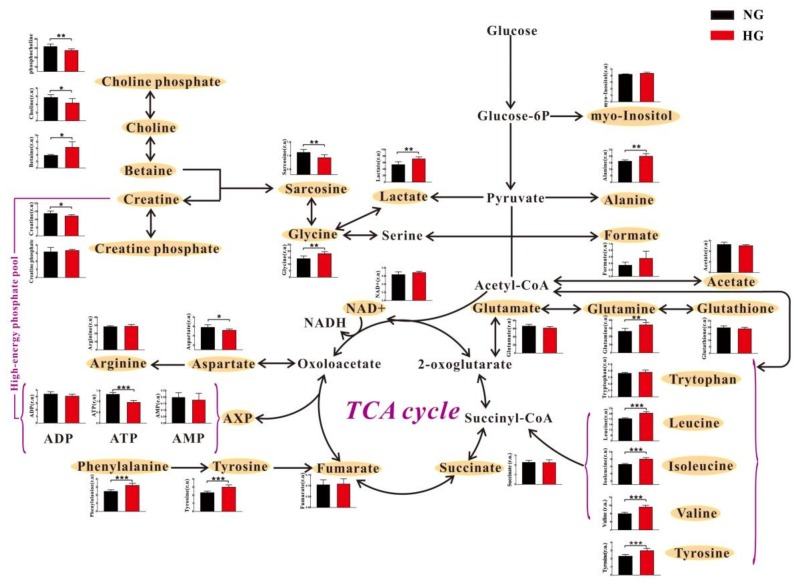
Metabolic changes in H9c2 cells under high glucose (HG, 33 mM) and normal glucose (NG, 5.5 mM) conditions: Data were expressed as Means ± SE (*n* = 6); r.u., relative unit; significant level: * *p* < 0.5; ** *p* < 0.01; *** *p* < 0.001.

These metabolites were relatively quantified and analyzed by pathway analysis, as illustrated in Figure 4. We found that the intracellular levels of BCAAs (leucine, isoleucine and valine) were significantly increased in H9c2 cells cultured under HG condition relative to NG condition. On the contrary, extracellular BCAAs levels were significantly reduced in HG culture medium as compared with NG culture medium (Table S1). This phenomenon was also observed in other amino acids, including alanine, tyrosine and phenylalanine. A significant lower level in choline phosphate was detected in the intracellular extracts of H9c2 cells under HG conditions than NG conditions (Figure 4). However, extracellular choline phosphate level was significantly increased in HG culture medium relative to NG culture medium (*p* = 0.0008), as shown in Table S1. For lactate and betaine, both intracellular and extracellular levels were significantly increased under HG conditions compared with NG conditions. In addition, relative to NG conditions, significant increases in glutamine and glycine as well as significant decreases in ATP, creatine, aspartate, sarcosine and choline were observed in the intracellular extracts of H9c2 cells under HG conditions (Figure 4). However, these metabolites were not detected or there was no significant difference in the extracellular extracts of H9c2 cells (Table S1).

### 3.3. High Glucose Induces Metabolic Changes in the Heart of Diabetic Rats

**Figure 5:**
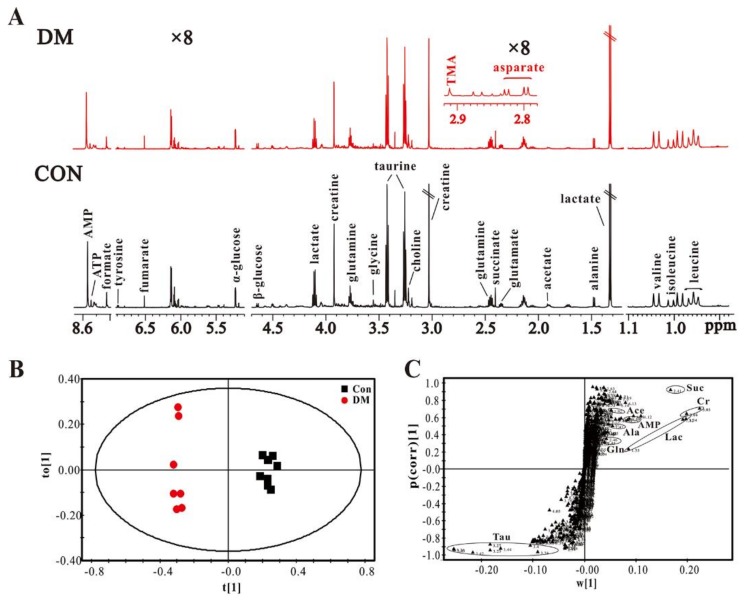
NMR-based cardiac metabolomic analysis: (**A**) typical 600 MHz ^1^H-NMR spectra obtained from the heart extracts in STZ-induced diabetic (DM) and normal control (Con) rats; OPLS-DA scores plot (**B)** and its corresponding S-plot (**C**) based on the heart metabolic profiles. Metabolite: Tau, taurine; Ace, acetate; AMP, adenosine monophosphate; Ala, alanine; Gln, glutamine; Suc, succinate; Cr, creatine; Lac, lactate.

To further examine HG-induced metabolic changes in vivo, heart tissues from STZ-induced T1D (DM) rats at eight weeks of age were analyzed by an NMR-based metabolomic approach. Figure 5A shows typical ^1^H-NMR spectra obtained from heart tissues in DM and normal control (Con) rats. A series of cardiac metabolites were identified, which involve amino acid metabolism (leucine, isoleucine, valine, glutamate, glutamine, taurine and glycine), energy metabolism (lactate, alanine, acetate, succinate, fumarate, creatine, ATP and AMP) and membrane metabolism (choline). The OPLS-DA model shows that DM rats can be clearly separated from Con rats based on the cardiac metabolome (Figure 5B). It can be seen from Figure 5C that an increase in taurine and decreases in acetate, alanine, succinate, creatine, lactate, AMP and glutamine in the heart of DM rats mainly contributed to this separation.

**Figure 6:**
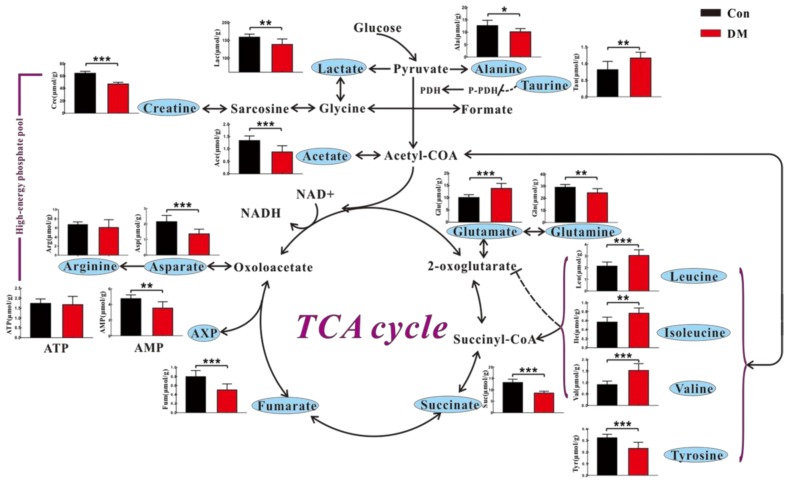
Metabolic changes in the heart of STZ-induced diabetic (DM) and normal control (Con) rats: Data were expressed as Means ± SE (*n* = 6); r.u., relative unit; significant level: * *p* < 0.5; ** *p* < 0.01; *** *p* < 0.001.

Figure 6 illustrates the result of metabolic pathway analysis in accordance with NMR-based cardiac metabolome in DM and Con rats. Similar to metabolic changes in vitro, we found that DM rats had higher levels of leucine, isoleucine and valine as well as lower levels of creatine and asparate in the heart than Con rats. However, significantly decreased levels of alanine, tyrosine and lactate were detected in the heart of DM rats, which is different from the in vitro results. As shown in Figure 6, energy metabolism-related metabolites, such as AMP, succinate and fumarate, were significantly reduced in the heart of DM rats relative to Con rats. In addition, we also observed a decreased acetate level as well as increased taurine and glutamate levels in the heart of DM rats (Figure 6).

## 4. Discussion

Cardiomyocyte death is a common symptom in advanced-stage diabetic patients due to high glucose (HG) stress. As expected, in the present study, we found that HG can promote cardiomyocyte death in both in vitro and in vivo studies. Therefore, we explored metabolic mechanisms underlying this phenomenon using an NMR-based metabolomic approach. Results show that HG-induced cardiomyocyte death may be implicated in energy metabolism, amino acid metabolism, cell membrane metabolism and osmoregulation.

Energy metabolism plays a critical role in maintaining normal heart function [42,43]. It is well known that glucose is the main substrate for energy metabolism. Glucose can be firstly converted to pyruvate, and then pyruvate is oxidized for ATP production through tricarboxylic acid (TCA) cycle or transformed into lactate by anaerobic glycolysis and alanine by transamination [20]. Although a high glucose level exists in the diabetic heart, glucose oxidation is impaired [44]. In this study, a significant decrease in ATP production was observed in HG-treated cardiomyocytes relative to normal controls, indicating a reduced energy metabolism under HG condition. Pointon et al. [45] reported that decreased cardiac ATP level could be a general feature of heart failure in mice. Moreover, Beer et al. [46] also found that myocardial ATP content was reduced by 35% in patients with dilated cardiomyopathy relative to healthy subjects using ^31^P magnetic resonance spectroscopy. Furthermore, a decrease in energy metabolism was confirmed in an in vivo study, as indicated by decreased levels of TCA intermediates (succinate and fumarate) and AMP in the heart of DM rats. However, there was no significant difference in cardiac ATP level between Con and DM rats. Of note, ATP measurement is significantly susceptible to tissue harvesting and extraction methods [47]. Thus, a change in cardiac ATP level is uncertain and needs to be further validated. Creatine is a key component of the creatine kinase phosphagen system that maintains energy homeostasis in the heart [48]. In this study, we found that creatine level was significantly reduced in cardiomyocytes under HG condition relative to NG condition. For in vivo studies, a decrease in creatine was also observed. Nascimben et al. [49] and Neubauer et al. [50] reported that heart failure patients also had a reduced myocardial creatine level. Taken together, these findings reveal that HG-induced cardiomyocyte death could be accompanied by a reduced energy metabolism. In our previous study, DM rats also had a lower energy metabolism in serum metabolome than Con rats, as indicated by decreased levels of citrate and creatine [51]. In fact, Nakagawa et al. [52] have reported that intracellular energy metabolism is a key factor in the regulation of cardiomyocyte death. Eguchi et al. [53] and Leist et al. [54] also proposed that intracellular energy level determines cell death fate. Hence, we further confirmed the role of energy metabolism on cardiomyocyte death using metabolomics both in in vitro and in vivo studies.

In the heart, amino acids serve as both important nutrients and signaling molecules [55]. Asparate as a non-essential amino acid is directly derived by transamination from a TCA cycle intermediate, oxaloacetate. Thus, in the present study, a reduced asparate level both in vitro and in vivo under HG conditions could be attributed to a defect of energy metabolism. Birsoy et al. [56] reported that asparate plays an important role in the mitochondrial electron transport chain for cell proliferation. Of note, the protective effect of asparate on cardiac function has been reported by Sivakumar et al. [57,58]. However, excess levels of branched-chain and aromatic amino acids (BCAAs and AAAs) would increase the risk for future diabetes via insulin resistance [59]. Magnusson et al. [23] also identified BCAAs and AAAs as novel markers of diabetes-induced cardiovascular disease (CVD) based on a 12-year follow-up clinical study. In this study, we found that BCAAs (leucine, isoleucine and valine) were significantly increased both in vitro and in vivo models under HG condition. To a certain extent, therefore, our finding supports the result of Magnusson et al. [23] from cellular and animal models. Moreover, AAAs (phenylalanine and tyrosine) were significantly increased only in vitro model under HG conditions; however, for the in vivo model, tyrosine level was significantly reduced under HG conditions and phenylalanine was not detected in this study. Our previous study based on serum metabolome revealed that DM rats had significantly higher levels of isoleucine and valine as well as significantly lower levels of phenylalanine and tyrosine as compared with Con rats [51]. Amino acid metabolism has been associated with heart diseases; however, BCAAs were paid more attention [22]. In the present study, we suggest that other amino acids excepting BCAAs also need to be considered for the study of cardiomyocyte death.

Intact cell membrane is essential for the maintenance of cardiomyocyte structure and function. Choline plays a key role in phospholipid synthesis of cell membranes [60]. Moreover, choline also possesses a cardiac cytoprotective effect via stimulating cardiac M_3_ subtype muscarinic acetylcholine receptors and alleviating oxidative damage [61,62]. In this study, we found that choline and choline-phosphate levels were significantly reduced in HG-treated cardiomyocytes relative to normal controls, indicating a disruptive membrane metabolism under HG conditions. In serum metabolome, relative to Con rats, we also found a significantly reduced choline level in DM rats [51]. Betaine is a derivative of choline by oxidation. This study reveals that a significant increase in betaine level was observed in HG-treated cardiomyocytes. Betaine also shows a protective effect on cardiomyocytes [63], but the role of choline cannot be replaced [64]. Thus, we speculate that a disordered choline-betaine metabolism could be responsible for HG-induced cardiomyocyte death. For in vivo study, this phenomenon was not detected using NMR-based metabolomics, while a significantly higher taurine level was found in the heart of DM rats than Con rats. Taurine is involved in a number of important physiological roles in the heart, such as osmoregulation, antioxidant activity and membrane stabilization [65]. Thus, taurine has been reported to protect diabetic rats against heart disease [66,67]. In the present study, an increased taurine level in the diabetic heart may reflect stress response and self-protection.

In addition, several metabolites exhibited inconsistent variations in vitro and in vivo. For example, relative to Con rats, levels of alanine, lactate and glutamine were reduced in the heart of DM rats, while this finding is contrary to the in vitro results. Glutamate level was significantly higher in the heart of DM rats than Con rats, but no significant difference was observed in vitro study. Thus, metabolic differences between in vitro and in vivo studies should be paid attention. The differences between in vitro and in vivo results may be attributed to several aspects: on the one hand, H9c2 cells as rat heart embryonic myoblasts possess skeletal muscle properties, but rat heart is an organ composed of various cell types; on the other hand, HG-induced cardiomyocyte changes in vitro cannot closely mimic the biological processes occurring in DM rats. The main limitation of an in vitro model is the lack of real physiological environment in living organisms, while its advantage is to exactly reflect cellular metabolism under a relative simple condition. Therefore, a combined in vitro and in vivo study will lead to a better understanding of molecular mechanisms of diseases.

## 5. Conclusions

In this study, both in vitro and in vivo studies show that HG promoted cardiomyocyte death. Metabolic changes underlying this phenomenon were investigated through an NMR-based metabolomic method. Several metabolites exhibited inconsistent variations in vitro and in vivo studies, but a series of common metabolic changes were also identified. For example, increases in branched-chain amino acids (BCAAs) and decreases in aspartate and creatine were observed under HG conditions, as compared with normal controls. In addition, energy metabolism-related metabolites, such as ATP, AMP, fumarate and succinate, were significantly reduced under HG conditions. Thus, we speculate that HG-induced cardiomyocyte death could be implicated in a decreased energy metabolism and an increased BCAA metabolism. Of note, based on the present study, further works need to be considered: (1) this finding should be verified in other cell lines and diabetic animal models; (2) a multi-analytical platform needs to be used for achieving a more detailed metabolic analysis; and (3) key enzymes or proteins involved in these metabolic pathways need to be further explored in order to facilitate understanding of potential mechanisms underlying HG-induced cardiomyocyte death.

## Supplementary Materials

Supplementary materials are available on line.

## Abbreviations

AAAs	aromatic amino acids
ADP	adenosine diphosphate
AMP	adenosine monophosphate
ATP	adenosine triphosphate
BCAAs	branch chain amino acids
DCM	diabetic cardiomyopathy
DM	diabetes mellitus
HE	haematoxylin-eosin
HG	high glucose
NAD+	nicotinamide adenine dinucleotide
NG	normal glucose
NMR	nuclear magnetic resonance
OPLS-DA	orthogonal partial least squares-discriminant analysis
STZ	streptozotocin
TCA	tricarboxylic acid
