# Supplementary material for: High Glucose-Induced Cardiomyocyte Death May Be Linked to Unbalanced Branched-Chain Amino Acids and Energy Metabolism
Source: Molecules. 2018 Apr 1;23(4):807. doi: 10.3390/molecules23040807 (PMC6017930; doi:10.3390/molecules23040807)
Supplement: Supplementary file 1 [file molecules-23-00807-s001.pdf]

High glucose-induced cardiomyocyte death may be linked to unbalanced branched-chain amino acids and energy metabolism

Xi Zhang[a], Qiuting Lin[a], Jiuxia Chen[a], Tingting Wei[a], Chen Li[a], Liangcai Zhao[a], Hongchang Gao[a,*] and Hong Zheng[a,*]

[a] Institute of Metabonomics & Medical NMR, School of Pharmaceutical Sciences, Wenzhou Medical University, Wenzhou 325035, China

*Corresponding author: Tel.: +86 577 86699715; E-mail: gaohc27@wmu.edu.cn (H.C. Gao); 123zhenghong321@163.com (H. Zheng).

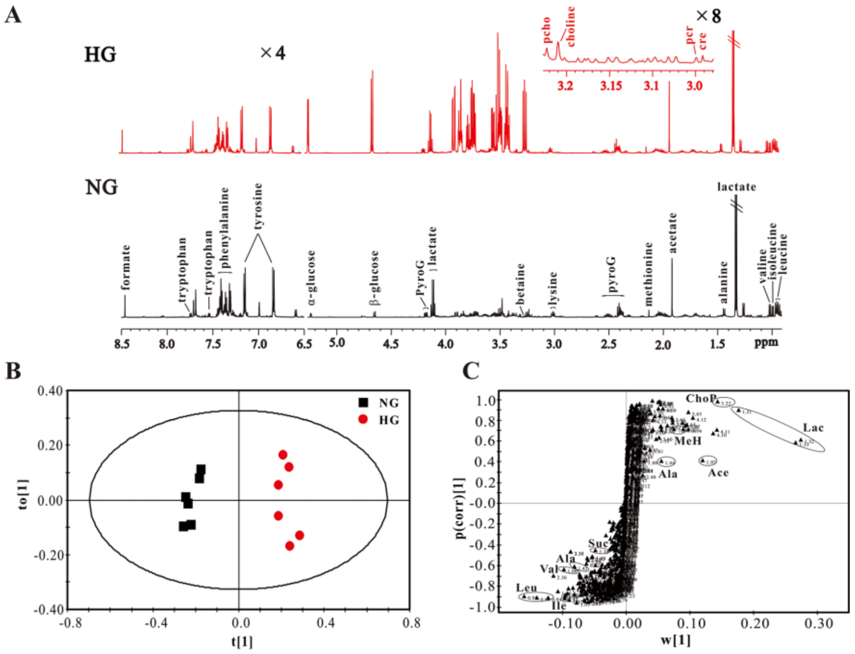

Fig. S1. NMR-based metabolomic analysis: (A) Typical 600 MHz ^{1}H NMR spectra obtained from the extracellular extracts under high glucose (HG, 33 mM) and normal glucose (NG, 5.5 mM) conditions; OPLS-DA scores plot (B) and its corresponding S-plot (C) based on the metabolomic data of the extracellular extracts. Metabolite: Leu, leucine; Val, valine; Ala, alanine; Suc, succinatte; ChoP, choline phosphate; Lac, lactate; MeH, methionine; Ace, acetate.

Formatted: English (Australia)

Table S1. Changes in metabolite levels from the extracellular extracts under normal glucose (NG) and high glucose (HG) conditions.

δ^1H(ppm)	Metabolite	NG	HG	P value
0.96	leucine	22.76 ±0.92[a]	20.52 ±0.90	0.0017
0.94, 1.01	isoleucine	17.06 ±0.66	14.97 ±0.74	0.0004
0.99, 1.04	valine	18.35 ±0.57	16.58 ±0.47	0.0002
1.33, 4.12	lactate	200.76 ±15.18	232.29 ±19.9	0.0117
1.48	alanine	18.60 ±0.60	17.89 ±0.53	0.0538
1.91	acetate	34.83 ±4.18	38.04 ±3.26	0.1693
2.14	methionine	4.07 ±0.14	3.57 ±0.13	0.0001
3.03, 3.92	creatine	0.80 ±0.02	0.76 ±0.08	0.2013
3.05, 3.95	creatine phosphate	0.82 ±0.04	0.67 ±0.04	0.0001
3.21	choline	3.01 ±0.18	2.97 ±0.17	0.6917
3.22	choline phosphate	1.82 ±0.07	2.11 ±0.13	0.0008
3.30	betaine	1.38 ±0.08	1.89 ±0.07	0.0000
6.91	tyrosine	0.53 ±0.02	0.48 ±0.03	0.0029
7.38	phenylalanine	7.04 ±0.19	6.56 ±0.10	0.0002
7.55	tryptophan	0.54 ±0.02	0.53 ±0.02	0.8043
8.46	formate	0.61 ±0.05	0.60 ±0.07	0.8282

[a] Metabolite level was calculated in accordance with the peak area by reference to the internal TSP concentration and expressed as Means ± SE (n = 6).
